# Evaluation of the effects of chitosan application and growing media on the adaptation process of fercal grape rootstock

**DOI:** 10.1186/s12870-025-07197-z

**Published:** 2025-08-28

**Authors:** Kevser Yazar

**Affiliations:** Agriculture Faculty Horticulture Department, University of Selcuk, 42130 Konya, Türkiye

**Keywords:** Bio-stimulant, Foliar treatment, Growing media, Grapevine rootstock, Plantlet survival

## Abstract

**Background:**

In plant micropropagation, the adaptation phase represents one of the most critical limiting steps due to the high mortality rates commonly observed under ex-vitro conditions. The Fercal grapevine rootstock, characterized by its high adaptability to calcareous soils, holds significant potential for large-scale propagation. Therefore, evaluating strategies to improve survival during the transition from in vitro to ex-vitro environments is essential. This study investigates the influence of different growing media and foliar-applied chitosan doses on the adaptation success of Fercal rootstock plantlets.

**Results:**

After transferring the plantlets to media containing either cocopeat or black sand, foliar chitosan treatments were applied at concentrations of 0, 15, and 30 mg L^−1^. Morphological and physiological parameters such as survival rate (%), shoot length (cm), shoot diameter (mm), internode length (cm), leaf area (cm²), number of nodes (per plant), number of leaves (per plant), and chlorophyll content (SPAD value) were systematically evaluated. The results showed that chitosan treatments significantly improved survival compared to the control group, which exhibited complete mortality (0% survival) in both media. The highest survival rate, 66.66%, was obtained with 30 mg L^−^¹ chitosan in black sand, while the greatest shoot elongation (24.63 cm) was observed in cocopeat treated with 15 mg L^−^¹ chitosan. Morphological responses varied depending on both the chitosan concentration and the type of growing medium. Principal component analysis and hierarchical cluster analysis revealed strong correlations among internode length, shoot number, and survival rate.

**Conclusion:**

Chitosan is a promising bio-stimulant for enhancing the ex-vitro performance of grapevine rootstocks, with its effectiveness strongly influenced by the physicochemical characteristics of the growing substrate. The combined use of inert substrates with high chitosan doses or organic substrates with moderate doses resulted in optimal outcomes. These findings provide a basis for refining micropropagation protocols, especially under environmentally challenging conditions.

## Introduction

Grapevines (*Vitis* spp.) are widely cultivated due to their economic significance and diverse applications in wine, table grape and raisin production. According to FAOSTAT (2023), the global vineyard area is approximately 6,595,680 hectares, with an annual grape production of about 72.5 million tons. Turkey ranks sixth worldwide in vineyard area, covering approximately 377.848 hectares, and seventh in grape production with an annual yield of nearly 3.4 million tons (FAO, https://www.fao.org/faostat). These statistics highlight the strategic importance of viticulture in Turkey and emphasize the need for efficient propagation and adaptation strategies to support the industry. In this context, achieving sustainable and high-quality grape production depends largely on the use of rootstocks that are resistant to phylloxera, well adapted to diverse ecological conditions and exhibit good graft compatibility with commercial grapevine cultivars [1].

Soils in Central Anatolia, including the Konya region, are predominantly calcareous with high pH and low organic matter content. According to Eyüpoğlu [2] and Akay et al. [3], over 63% of Turkish soils have a pH above 7.5, and nearly 59% contain more than 5% calcium carbonate (CaCO_3_), conditions which significantly limit the availability and uptake of essential nutrients such as phosphorus, iron, and zinc. Among the rootstocks recommended for use in vineyards with calcareous soils, Fercal stands out due to its high tolerance to lime [4]. Therefore, the propagation of Fercal rootstock is of particular importance for viticultural practices on calcareous soils. The use of clonal propagation techniques for the multiplication of vigorous and disease-free rootstock genotypes plays a critical role in meeting the demands of modern viticulture [5]. Plant tissue culture offers several advantages, including virus elimination, improved disease resistance, sterile propagation conditions, and rapid multiplication [6–9]. However, plant tissue culture also has certain disadvantages, such as high initial costs, the need for skilled labor, somaclonal variation, and the risk of microbial contamination if aseptic conditions are not strictly maintained [10, 11]. Special protocols developed especially for grapevine, which are among the economically important species, allow these advantages to be evaluated effectively [12–14].

Acclimatization of plants propagated by tissue culture constitutes one of the most critical stages of tissue culture [15]. The success of ex-vitro adaptation varies considerably depending on plant species and the treatments applied. In *Oncidium baueri*, a survival rate of 62.5% was achieved [16]. For Fercal grapevine rootstock, survival increased from 0% (control) to 66% with 1000 µL ortho silicon, and in 41B, it reached 100% [17]. In *Stevia rebaudiana*, pre-acclimatization with 100 mg/L BA enhanced survival from 60.5–75.8% [18]. The low success rate is affected by the interaction between physiological, morphological and environmental characteristics, as well as the methods used for acclimatization [19]. Factors such as fungal infections that occur in plants or in the growing environment during acclimatization, reduced photosynthetic efficiency due to insufficient optimization of leaves to external conditions, and excessive water loss affect viability and plant quality [8, 20, 21]. To reduce the effects of these stress factors, it is important to apply different methods of acclimatization [17, 22–24].

Plantlet survival and growth during acclimatization are largely determined by the physicochemical properties of the ex-vitro culture medium [25, 26]. The use of substrates derived from organic sources, such as coconut husk, has been shown to enhance root development and facilitate plant adaptation due to their favorable structure and high water retention [27, 28]. For instance, survival rates reached 62.5% in *Oncidium baueri* using a carbonized rice husk (CRH) and coconut fiber (CF) mixture [16], and 95.8% in *Campomanesia* seedlings grown in a soil coconut husk substrate [29]. However, organic materials may also exhibit unfavorable characteristics such as high salinity, low pH, or microbial imbalance, which can promote the development of plant pathogens [30, 31]. In contrast, inorganic materials offer several advantages in acclimatization, including the supply of mineral nutrients, provision of appropriate physical structure and aeration, ease of sterilization, and chemical stability [27, 32]. Black sand (ReeFlowers Iceland Black Sand, 1–2 mm, https://www.reeflowers.com) is a chemically inert and 100% natural substrate. During its preparation, activated carbon and ultrafiltration techniques were used to reduce organic impurities and improve substrate quality. It does not alter general hardness (gH), carbonate hardness (kH), or pH, making it a stable and clean medium suitable for use in plant acclimatization studies. While black sand has been used as an inert substrate in ex-vitro adaptation and acclimatization studies [17], its comparative evaluation with organic materials has not yet been conducted. Therefore, assessing its performance alongside organic substrates is important to determine its potential effectiveness in improving ex-vitro acclimatization.

Chitosan is a deacetylated derivative of chitin, a natural polysaccharide commonly obtained from crustacean shells such as crabs, and can be solubilized through alkaline or enzymatic processes [33]. Chitosan has been extensively studied for its effects on phenolic compound accumulation, plant metabolism, and growth regulation [34]. It enhances the biosynthesis of secondary metabolites and activates defense-related pathways that improve resistance to pathogens and pests [35, 36]. In addition, chitosan enhances seed germination, root–shoot growth, and stress tolerance in various plant species. For example, it improved germination and seedling emergence in bell pepper (*Capsicum annuum* L.) under cold stress [37], and promoted root–shoot development and drought tolerance in grapevine (*Vitis vinifera* L.) under water deficit [38]. In tissue culture techniques, the acclimatization success of plantlets is as crucial as the number of plantlets produced. For instance, in lantana (*Lantana camara* L.), chitosan improved in vitro shoot regeneration and ex-vitro survival [39], while in banana (*Musa* spp.), it enhanced growth, water status, and antioxidant activity, leading to higher survival rates during acclimatization [40].

Despite advancements in micropropagation, acclimatization remains a key challenge due to low survival rates and environmental stress sensitivity [41]. Improving this stage depends on selecting appropriate substrates and bio-stimulants. Black sand offers advantages such as inertness and ease of sterilization (https://www.reeflowers.com/), while chitosan is known to enhance stress tolerance in plants [42]. This study aims to investigate the influence of different growing media (cocopeat and black sand) and foliar chitosan applications (0, 15, and 30 mg L^−^¹) on the ex-vitro adaptation efficiency of Fercal grapevine rootstock.

## Materials and methods

### Plant Material and *In Vitro* Propagation Methods

In this study, the Fercal grapevine rootstock [Berlandieri Colombard 1B × Richter 31) https://www.vivc.de/], known for its high resistance to phylloxera, nematodes, and cotton root rot, and well-adapted to calcareous soils with high pH levels, was selected as the plant material. Rootstock cuttings were obtained through the Sapling Producers Sub-Association (https://www.fuab.org.tr/) and provided as certified plant material in accordance with Turkish Standards TS 3981 and TS 3912 (https://www.tse.org.tr/). TS 3981 defines the quality standards for grapevine plants propagated vegetatively from *Vitis vinifera* L. and hybrid varieties, while TS 3912 outlines the general characteristics, sampling procedures, and testing methods for cuttings, rootstocks, and scions. The cuttings were initially rooted in the greenhouse and subsequently transplanted into the grapevine rootstock parcel of the Department of Horticulture, Faculty of Agriculture, Selçuk University (38.03507° N, 32.50114° E; Konya, Türkiye), where the resulting plants were used in the experimental study. As no wild plant collection was involved in this study, no specific permissions, formal identifications, or voucher specimens were required. During the active growth phase, single node microcuttings were excised from rootstock plants grown in the grapevine rootstock parcel of Selçuk University [43, 44]. The explants were disinfected under aseptic conditions in a vertical laminar flow cabinet by sequential treatment with 70% ethanol (2 min), 12% sodium hypochlorite (NaOCl) (15 min), and then rinsed three times with sterile distilled water. After surface sterilization, micro cuttings were placed in jars containing Murashige and Skoog (MS) culture medium supplemented with 3% sucrose, 0.7% agar, and 1 mg L^−1^ BAP (Benzylaminopurine) [44]. The cultured explants were maintained under controlled environmental conditions at 25 ± 1 °C, with a 16-hour light/8-hour dark cycle and a light intensity of 4000 lx m^−2^ [22, 44]. In the rooting stage, Murashige and Skoog (MS) medium was supplemented with 1 mg L^−1^ IBA (Indole-3-butyric acid).

### Ex-vitro adaptation process and experimental design

**Fig. 1:**
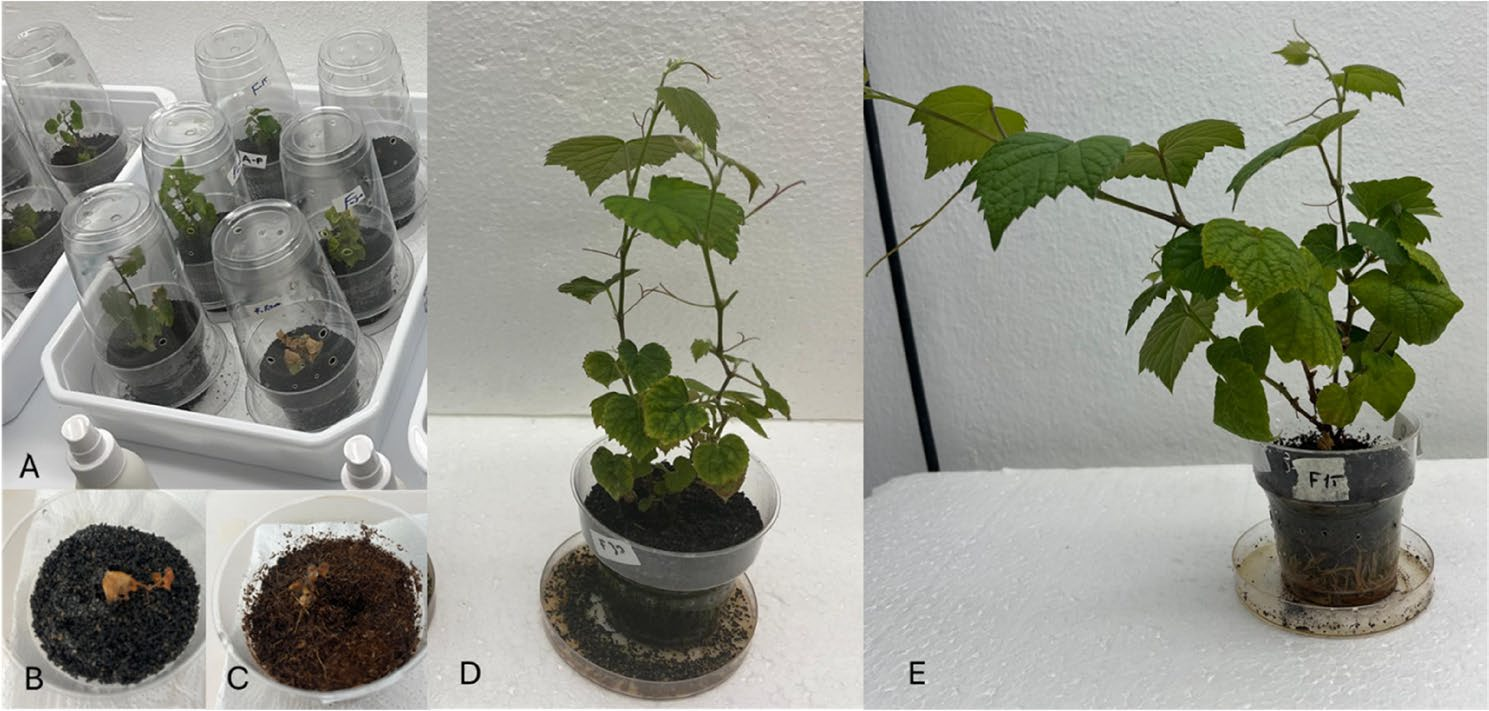
Foliar chitosan applications during the adaptation phase and representative plants from the corresponding treatment groups. **A** Adaptation setup in which foliar chitosan treatments were applied at 5-day intervals for a total duration of 15 days. **B**–**C** Plants in the control groups grown in black sand (**B**) and coco peat (**C**) did not survive during the adaptation phase. **D** A representative plant from the group grown in black sand and treated with 30 mg L^−1^ chitosan through foliar application. **E** A representative plant from the group grown in coco peat and treated with 15 mg L^−1^ chitosan through foliar application

**Fig. 2:**
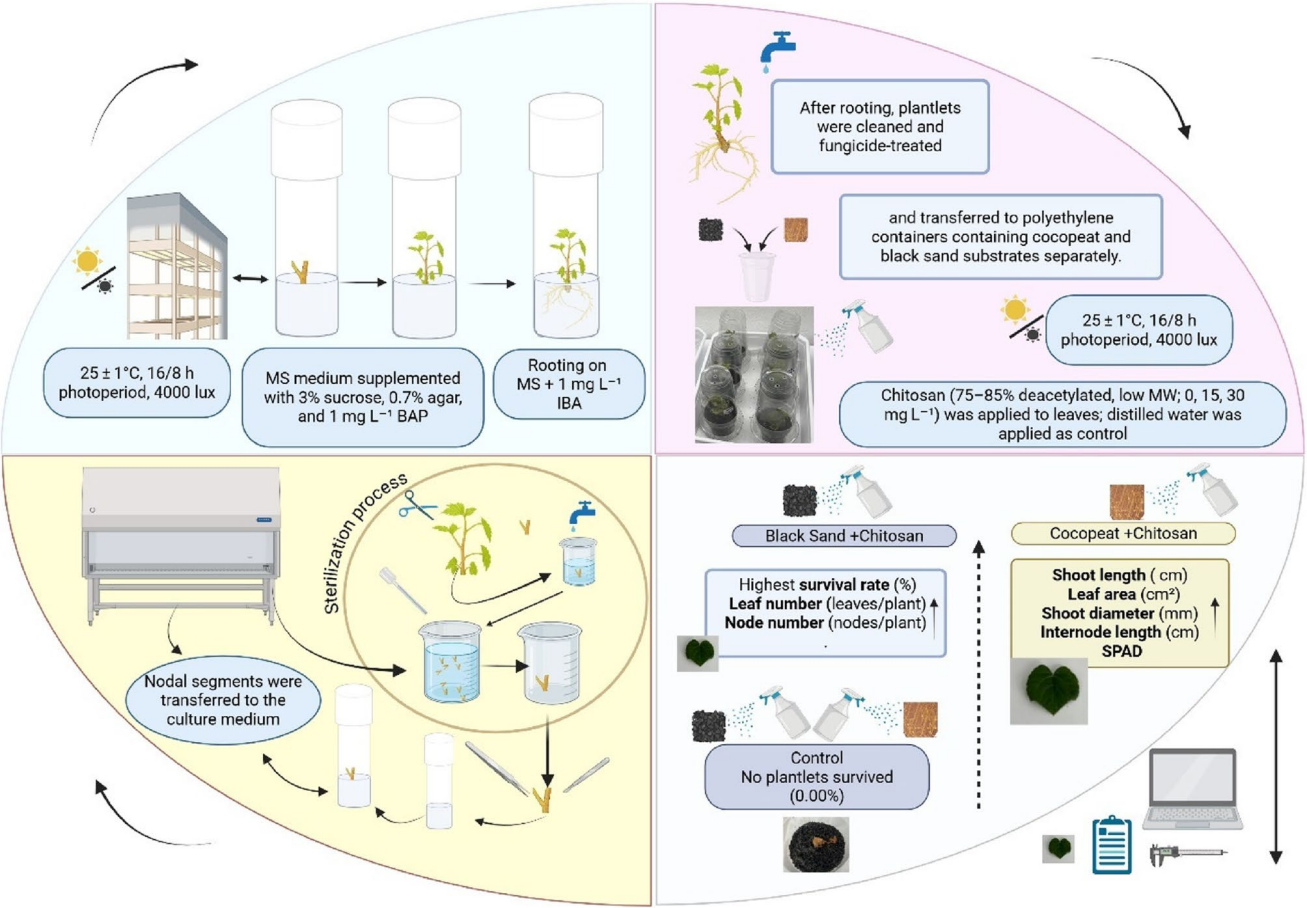
The experimental design and graphical summary of the results illustrating the effects of foliar-applied chitosan and different growing media on the ex-vitro adaptation performance of in vitro-derived plantlets were created using BioRender (BioRender https://www.biorender.com/)

Once root development was observed, plantlets were washed with lukewarm water to remove the culture medium, treated with a fungicide solution, and transplanted into polyethylene containers filled with either cocopeat or black sand, depending on the treatment. Subsequently, low molecular weight chitosan with a 75–85% degree of deacetylation (Sigma-Aldrich; C3446) was dissolved in 2% (v/v) lactic acid prepared using (S)-Lactic Acid, ~ 90% (Sigma-Aldrich; CAS No: 79-33-4; https://www.sigmaaldrich.com). The pH of the solutions was adjusted to 5.6 with 1 N NaOH prior to application [45], and the solutions were immediately applied to the leaves at concentrations of 0, 15, and 30 mg L^−1^. Distilled water was used as control (Figs. 1 and 2). The plantlets were transferred into polyethylene containers filled with the respective growing media, which had been previously autoclaved at 121 °C for 20 min to ensure sterilization. As shown in Fig. 1-A, during the adaptation process, these containers were covered with inverted larger polyethylene containers to maintain high humidity. To facilitate gradual adaptation to ex-vitro conditions, small holes were progressively opened in the covers of the outer containers during the first 10 days of adaptation. The adaptation was carried out in a climate chamber under controlled environmental conditions at 25 ± 1 °C, with a 16-hour light/8-hour dark cycle and a light intensity of 4000 lx m^−2^, and this environment was maintained throughout the 50-day adaptation period. Foliar chitosan applications were performed at concentrations of 0, 15, and 30 mg L^−1^, and were repeated every 5 days over a 15-day period. The experiment was designed according to a completely randomized design, including three replicates in each treatment, each consisting of 15 plantlets.

### Morphological and physiological parameters

The response of the plantlets to the treatments was determined based on several morphological and physiological parameters, including survival rate (%), number of nodes per plant, internode length (mm), shoot length (cm), number of leaves per plant, shoot diameter (mm), leaf area (cm²), and chlorophyll content (SPAD value). Survival rate was recorded 20 days after the beginning of adaptation, while all other parameters were measured on the 50th day in the plants that survived. In the measurement of morphological and physiological parameters, the [Cocopeat + chitosan (30 mg L^−1^)] treatment, which had the lowest survival rate, was taken as a reference to ensure consistency across all treatments and to maintain the structure of biological replication. Each treatment was evaluated with three replicates, and measurements were performed on four randomly selected viable plants per replicate (*n* = 12). The number of measured plants was standardized based on the treatment with the lowest survival rate, allowing for balanced comparisons among treatments despite differences in overall survival rates. The survival rate was expressed as the percentage of plantlets that survived adaptation, calculated by dividing the number of viable plants by the total number of transferred plantlets. The number of nodes and leaves was determined through direct counting for each plantlet. Shoot length and diameter were measured using a digital caliper [17]. For leaf area calculation, scanned leaf images were analyzed via Photoshop Portable Sfx. program. Soil Plant Analysis Development (SPAD) was estimated using a Minolta SPAD-520 m. Measurements were taken from the third and fourth fully expanded leaves located below the shoot apex on each surviving plant. For each leaf, readings were taken from opposite sides of the leaf blade, and the average value was recorded as the SPAD reading for that plant. SPAD measurements were conducted once, at the end of the 50th day, on a total of 12 plants per treatment [46, 47] (Figs. 1 and 2).

#### Data analysis

The experimental data were statistically analyzed using Tukey’s HSD test (*p* < 0.05) in JMP version 13 to identify significant differences among treatments. Interaction effects between chitosan application doses and growing media were also examined. Following the interaction analysis, comparisons were conducted separately for chitosan doses and growing media. Additionally, hierarchical cluster analysis (HCA) and principal component analysis (PCA) were performed using R software (v4.1.1, R Foundation for Statistical Computing) to further explore the correlations among variables.

## Results and discussion

### Survival rate (%)

The obtained results showed that the survival rates of the plantlets were significantly affected by the treatments (*p* < 0.05). Plantlets grown in both cocopeat and black sand media without chitosan application (control) were unable to maintain viability during the adaptation period (0.00%). In the cocopeat medium, the highest survival rate was recorded with 15 mg L^−1^ chitosan (53.34%), whereas in the black sand medium, the best result was obtained with 30 mg L^−1^ chitosan (66.66%). The highest survival rate overall (66.66%) was achieved with 30 mg L^−1^ chitosan in the black sand medium, which may suggest a synergistic interaction between the biological effect of chitosan and the physical properties of the black sand. In contrast, the same dose applied in the cocopeat medium resulted in a lower survival rate (33.33%). When medium means were considered, black sand yielded a higher average survival rate (37.71%) compared to cocopeat (28.89%). Regarding application means, all chitosan treatments (50.00%) positively influenced the survival rate compared to the control group (0.00%) (Table 1). These results indicated that the effectiveness of chitosan may vary depending on the characteristics of the growing medium. The findings strongly support the potential use of chitosan as a bio-stimulant during the adaptation phase.

**Table 1:** Effects of growing medium and Chitosan applications on adaptation success and plantlet development during ex-vitro transfer *

Parameter	Growing Medium	Chitosan Dose (mg/L^−1^)	Value	Growing Medium Mean	Application Mean
Survival Rate (%)	Cocopeat	0	0.00 ± 0.00e	28.89 *B*	0.00 C
		15	53.34 ± 0.32b		50.00 A
		30	33.33 ± 0.35d		50.00 A
	Black sand	0	0.00 ± 0.00e	37.71 *A*	0.00 C
		15	46.65 ± 0.31c		50.00 A
		30	66.66 ± 0.19a		50.00 A
Shoot Length (cm)	Cocopeat	0	-	22.89 *A*	-
		15	24.63 ± 0.74a		17.12 B
		30	21.15 ± 1.03b		19.63 A
	Black sand	0	-	13.86 *B*	-
		15	9.61 ± 0.63d		17.12 B
		30	18.12 ± 0.64c		19.63 A
Shoot Diameter (mm)	Cocopeat	0	-	1.85 *A*	-
		15	1.92 ± 0.03a		1.37 B
		30	1.76 ± 0.04b		1.48 A
	Black sand	0	-	1.03 *B*	-
		15	0.83 ± 0.06d		1.37 B
		30	1.22 ± 0.03c		1.48 A
Number of Leaves (per plant)	Cocopeat	0	-	7.01 *A*	-
		15	6.87 ± 0.06b		5.60 B
		30	7.15 ± 0.26b		8.57 A
	Black sand	0	-	7.17 *A*	-
		15	4.33 ± 0.58c		5.60 B
		30	10.00 ± 0.25a		8.57 A
Number of Nodes (per plant)	Cocopeat	0	-	7.76 *A*	-
		15	7.00 ± 0.06c		5.79 B
		30	8.52 ± 0.26b		9.28 A
	Black sand	0	-	7.31 *A*	-
		15	4.58 ± 0.58d		5.79 B
		30	10.03 ± 0.25a		9.28 A
Internode Length (cm)	Cocopeat	0	-	1.13 *A*	-
		15	1.03 ± 0.05b		0.91 B
		30	1.22 ± 0.01a		1.10 A
	Black sand	0	-	0.87 *B*	-
		15	0.78 ± 0.03c		0.91 B
		30	0.97 ± 0.02b		1.10 A
SPAD Value	Cocopeat	0	-	28.71 *A*	-
		15	28.98 ± 0.31a		27.74 B
		30	28.43 ± 0.45a		28.61 A
	Black sand	0	-	27.65 *B*	-
		15	26.50 ± 0.53b		27.74 B
		30	28.80 ± 0.46a		28.61 A
Leaf Area (cm²)	Cocopeat	0	-	25.10 *A*	-
		15	25.43 ± 0.33a		13.95 B
		30	24.76 ± 0.45a		19.01 A
	Black sand	0	-	7.87 *B*	-
		15	12.59 ± 0.52b		13.95 B
		30	3.15 ± 0.15c		19.01 A

Uppercase letters represent application means

Italic uppercase letters indicate growing medium means

*Different lowercase letters indicate significant differences in the interaction (p <0.05, Tukey test)

Acclimatization is a critical stage in plant tissue culture, where plantlets are highly vulnerable to environmental stressors and microbial infections [15]. Control plantlets were unable to survive in either cocopeat or black sand media, highlighting the critical role of chitosan as a supportive treatment in enhancing plantlet survival during the acclimatization stage. While cocopeat can promote root development due to its high-water retention, it may also create excess moisture and microbial imbalance, which can impair survival despite [30, 31]. In contrast, black sand (https://www.reeflowers.com/), offered a more favorable environment, particularly when combined with chitosan. Chitosan treatment significantly improved survival rates across both media, with the highest success observed at 30 mg L^−1^ in black sand. Beyond its bio-stimulant role, chitosan’s well-documented antimicrobial properties likely contributed to reducing pathogenic pressure during acclimatization [33, 35]. The results suggest that the interaction between a microbially stable substrate and chitosan application provides a synergistic effect, enhancing plantlet overall ex-vitro adaptation performance.

### Shoot length (cm)

Shoot length values varied significantly depending on the treatments (*p* < 0.05). In the cocopeat medium, the highest shoot length (24.63 cm) was recorded with the application of 15 mg L^−1^ chitosan, followed by 30 mg L^−1^ chitosan (21.15 cm). In the black sand medium, shoot lengths of 18.12 cm and 9.61 cm were obtained with 30 mg L^−1^ and 15 mg L^−1^ chitosan, respectively. These results indicate that 15 mg L^−1^ chitosan had a positive effect on shoot development in both media. However, the overall highest shoot length was observed in the cocopeat medium with 15 mg L^−1^ chitosan. When medium means were considered, cocopeat generally promoted greater shoot elongation (22.89 cm) compared to black sand (13.86 cm). Additionally, according to application means, 30 mg L^−1^ chitosan resulted in longer shoots than 15 mg L^−1^ in the black sand medium, while an opposite effect occurred in the cocopeat medium (Table 1).

Shoot length was notably influenced by the interaction between chitosan concentration and the type of growing medium, with the highest elongation recorded in cocopeat supplemented with 15 mg L^−1^ chitosan. Cocopeat, derived from coconut husk fibers, provides favorable physicochemical properties including high porosity, excellent water holding capacity, and a balanced supply of essential micronutrients such as iron, potassium, manganese, copper, and zinc [48]. These features support root aeration and facilitate cell expansion, ultimately promoting shoot growth [49–51]. Literature also supports the effectiveness of cocopeat in promoting shoot development during acclimatization in various species, including *Fragaria* × *ananassa* [51], and *Elaeis guineensis* [52]. The observed enhancement in shoot elongation under moderate chitosan concentration may reflect a synergistic effect between chitosan and cocopeat. Chitosan is a natural polysaccharide with biocompatible and biodegradable properties, and it has been reported to stimulate auxin-like signaling, enhance chlorophyll synthesis, and improve nutrient uptake. In *Mentha piperita* L., foliar application of irradiated chitosan at 80 mg L^−1^ increased shoot length by 39.5%, while higher concentrations suppressed growth, highlighting the dose-dependent nature of its effects [53]. Similarly, in *Lantana camara* L., low chitosan concentrations promoted shoot elongation, but concentrations above 1.0 mg L^−1^ caused stunted shoots and leaf necrosis [39]. In contrast, greater shoot elongation was also recorded in black sand medium treated with 30 mg L^−1^ chitosan. Black sand is an inert and nutrient-poor substrate, and under such conditions, the physiological effects of chitosan may become more pronounced due to the minimal background nutrient interference. Chitosan has been shown to activate antioxidant defense mechanisms, regulate stomatal function, and stimulate vascular development, all of which contribute to improved shoot growth even under suboptimal growing conditions [40]. These findings underline that both the physicochemical nature of the growing medium and the applied chitosan concentration are critical determinants of shoot elongation during the ex-vitro adaptation process.

### Shoot diameter (mm)

Shoot diameter values varied significantly depending on the treatments (*p* < 0.05). In the cocopeat medium, the highest shoot diameter (1.92 mm) was recorded with the application of 15 mg L^−1^ chitosan, followed by 30 mg L^−1^ chitosan (1.76 mm). In the black sand medium, shoot diameters of 1.22 mm and 0.83 mm were obtained with 30 mg L^−1^ and 15 mg L^−1^ chitosan, respectively. These results indicate that shoot diameter was generally higher in cocopeat than in black sand, regardless of the chitosan dose. However, 30 mg L^−1^ chitosan was more effective than 15 mg L^−1^ in enhancing shoot diameter in the black sand medium. When medium means were considered, cocopeat promoted greater shoot thickness (1.85 mm) compared to black sand (1.03 mm). Considering application means, 30 mg L^−1^ chitosan resulted in thicker shoots than 15 mg L^−1^ overall, while the highest individual shoot diameter was observed in cocopeat with 15 mg L^−1^ chitosan (Table 1).

Shoot diameter and shoot length increased in parallel depending on both chitosan concentration and growing medium, with the highest values observed in cocopeat supplemented with 15 mg L^−1^ chitosan. This finding suggests that a moderate dose of chitosan in an organic and porous substrate supports both vertical and radial shoot development by improving nutrient availability, promoting cell expansion, and facilitating vascular tissue formation [54, 55]. In *Solanum lycopersicum* L., application of 150 mg L^−1^ chitosan under salinity conditions led to an increase in stem diameter and shoot length [56], while in *Solanum tuberosum* L., 500 mg L^−1^ chitosan enhanced shoot fresh weight by approximately 42% and dry weight by 65%, confirming the positive effect of chitosan on biomass accumulation and stem thickening [57]. Likewise, in *Dendrobium* Shavin White orchids, the use of rice husk, peat moss, and oil palm EFB (Oil Palm Empty Fruit Bunch) in combination with 15 ppm chitosan increased plant height to 6.55% and leaf number to 4.14%, indicating that substrates with good aeration and chitosan application can synergistically promote structural shoot development [55]. In *Prunus davidiana* L. and *Vitis vinifera* L., increased shoot length, node number, and chlorophyll content under chitosan treatments reflect a coordinated growth pattern that also implies thickening of stem tissues including shoot diameter. In grapevine, for example, 1% Biochikol 020 PC increased shoot length by 71%, shoot number by 29%, and node number by 33% under drought conditions [58]. Similarly, in *Prunus davidiana*, application of carboxymethyl chitosan improved shoot biomass and chlorophyll concentration [59]. These structural and physiological enhancements indicate that chitosan can stimulate radial expansion through its effects on hormonal balance, nutrient transport, and vascular differentiation. Altogether, the interaction between optimized chitosan levels and substrate characteristics plays a critical role in regulating shoot diameter during acclimatization, as also demonstrated in various micropropagated species including orchids [27, 55].

### Number of leaves (per plant)

Leaf number per plant varied significantly depending on the treatments (*p* < 0.05). In the black sand medium, the highest number of leaves (10.00) was recorded with the application of 30 mg L^−1^ chitosan, followed by the control samples (7.17). In the cocopeat medium, leaf numbers of 7.15 and 6.87 were obtained with 30 mg L^−1^ and 15 mg L^−1^ chitosan, respectively. These results indicate that chitosan application, particularly at 30 mg L^−1^, promoted leaf development more effectively in the black sand medium. However, leaf numbers in the cocopeat medium remained relatively stable across chitosan doses, with values comparable to the control group (7.01). When medium means were considered, cocopeat supported a slightly higher average leaf number (7.01) than black sand (7.17). Considering application means, 30 mg L^−1^ chitosan resulted in the highest leaf production overall (8.57), while the lowest value was observed with 15 mg L^−1^ chitosan in the black sand medium (4.33) (Table 1).

Several studies have reported that chitosan can exert positive effects on plant growth, particularly by enhancing leaf formation. For example, in *Basella alba*, increasing concentrations of chitosan led to greater plant height and leaf number, with the highest values observed at 100 ppm [60]. In *Lactuca sativa*, soil-applied chitosan significantly enhanced leaf number, leaf area, fresh weight, and dry weight at concentrations of 0.05%, 0.10%, and 0.15%. However, application at 0.30% resulted in negative effects, emphasizing the importance of optimizing dosage [61]. In *Lycopersicon esculentum* under saline stress, 150 mg L^−1^ chitosan significantly increased average leaf number and other growth traits, likely due to enhanced photosynthetic area [56]. Similar promotive effects were reported in Freesia with high-molecular-weight chitosan [62], and in *Solanum tuberosum*, where chitosan improved leaf number at 30 and 50 days after tuber planting [63]. In the present study, leaf number was significantly affected by both chitosan concentration and the type of growing medium (*p* < 0.05). The highest number of leaves was observed in black sand treated with 30 mg L^−1^ chitosan, suggesting that the physiological effects of chitosan become more evident in nutrient-deficient substrates. In cocopeat, although chitosan application also resulted in statistically significant differences, the effect size was smaller. This outcome may be attributed to the inherently favorable structure and nutrient content of cocopeat, which supports vegetative growth without additional inputs. Supporting this, previous studies have shown that *Fragaria* × *ananassa* plantlets grown in pure cocopeat had high leaf number and leaf area even without any bio-stimulant application [51]. Similarly, in *Punica granatum* var. Bhagwa, cocopeat-enriched substrates promoted considerable leaf formation during acclimatization without the need for supplementary treatments [64]. Taken together, these findings support the hypothesis that chitosan, particularly when applied under suboptimal growing conditions, may promote leaf development and aid in plant acclimatization.

### Number of nodes (per plant)

Node number per plant varied significantly depending on the treatments(*p* < 0.05). In the black sand medium, the highest number of nodes (10.03) was observed with the application of 30 mg L^−1^ chitosan, followed by the control group (7.31). In the cocopeat medium, node numbers of 8.52 and 7.00 were recorded with 30 mg L^−1^ and 15 mg L^−1^ chitosan, respectively. These results suggest that chitosan application, particularly at the higher dose of 30 mg L^−1^, positively influenced node formation in both media. However, the effect was more pronounced in the black sand medium. In contrast, the control treatments in both media showed relatively high node numbers, indicating that the medium itself also played a significant role. When medium means were considered, cocopeat supported a slightly higher node number (7.76) than black sand (7.31). Considering application means, 30 mg L^−1^ chitosan resulted in the highest average number of nodes per plant (9.28), while the lowest was observed with 15 mg L^−1^ chitosan in black sand (4.58) (Table 1).

Node number per plant was notably influenced by both chitosan concentration and the type of growing medium, with the highest value observed in black sand treated with 30 mg L^−1^ chitosan. Similarly, in *Vitis vinifera*, a study demonstrated that the application of a 1% chitosan-based formulation (Biochikol 020 PC) increased internode number by 33%, shoot number by 29%, and shoot length by 71% compared to the control group [58]. In another experiment on the same species, application of 1.75% (v/v) formulated Chitogel under in vitro conditions significantly enhanced the number of nodes, which was accompanied by improvements in shoot elongation and dry biomass accumulation [65]. In this case, the average number of nodes per plant increased from 4.33 in the control group to 7.33 in the Chitogel-treated group, corresponding to an approximate 69% increase. In *Ipomoea aquatica*, treatment with 2500 kg ha^−1^ of cocopeat resulted in 10.3 leaves per plant, compared to 8.2 leaves in the control group. Given the positive correlation between leaf and node number, this finding suggests that cocopeat may indirectly promote nodal development [48]. Similarly, in *Dendrobium* Shavin White, the use of an organic medium containing oil palm EFB supplemented with 15 ppm chitosan led to an average of 4.14 leaves per plant, indicating enhanced phytomer activity and nodal initiation [55]. In *Musa acuminata*, 0.1% chitosan application increased the number of leaves from 6.7 to 7.7 on day 21 of acclimatization [28]. Additionally, in *Fragaria* × *ananassa* seedlings, cocopeat was reported to be the most effective substrate for leaf production among tested media [51]. Similarly, Similarly, in *Grammatophyllum scriptum*, sphagnum-based substrates demonstrated the broad compatibility of chitosan with diverse growing environments [66]. These findings collectively indicate that both chitosan applications and organic substrates can support nodal structure formation through improved leaf development and axial growth. Notably, although nodal enhancement was observed in both growing media, the highest node number (10.03) recorded in black sand supplemented with 30 mg L^−1^ chitosan suggests a more pronounced effect under inorganic and nutrient-poor conditions. This implies that in the absence of supportive properties typically provided by organic substrates, chitosan may more directly stimulate physiological responses and thus exert a stronger influence on developmental processes.

### Internode length (cm)

Internode length was significantly influenced by both the chitosan treatments (*p* < 0.05). In the cocopeat medium, the longest internode length (1.22 cm) was recorded with the application of 30 mg L^−1^ chitosan, followed by 15 mg L^−1^ chitosan (1.03 cm). In the black sand medium, internode lengths of 0.97 cm and 0.78 cm were obtained with 30 mg L^−1^ and 15 mg L^−1^ chitosan, respectively. These results indicate that increasing the chitosan concentration positively influenced internode elongation in both growing media, with more prominent effects observed in cocopeat. Moreover, the control samples showed lower mean internode lengths in both media, highlighting the contribution of chitosan to internodal development. When medium means were considered, cocopeat promoted longer internodes (1.13 cm) compared to black sand (0.87 cm). Considering application means, 30 mg L^−1^ chitosan resulted in the greatest internode elongation overall (1.10 cm), while the shortest internodes were observed with 15 mg L^−1^ chitosan in black sand (0.78 cm) (Table 1).

Internode length was significantly affected by both chitosan treatments and the type of growing medium, with the longest internodes observed in cocopeat treated with 30 mg L^−1^ chitosan. This suggests that increased chitosan concentration may stimulate internodal elongation, particularly when combined with substrates that offer better aeration and moisture retention. Similar effects have been documented in *Mentha piperita* L., where foliar application of 80 mg L^−1^ ionized chitosan increased stem length up to 93.3 cm and maximized both fresh and dry biomass accumulation [53]. In *Lycopersicon esculentum*, foliar application of 0.75 mg mL^−1^ chitosan under normal conditions improved shoot dry and fresh weights as well as leaf area [67]. However, the same study also noted that higher concentrations (0.75 and 1 mg mL^−1^) eventually reduced Fv/Fm values, indicating a potential inhibition of photosynthetic efficiency. In a separate study, foliar application of 150 mg L^−1^ chitosan significantly enhanced leaf number and area under salinity stress [56]. On the other hand, adverse effects on internodal length were noted in *Solanum tuberosum* L., where application of 500 mg L^−1^ soluble chitosan significantly reduced plant height and internodal spacing, possibly due to disrupted root development [57]. In contrast, lower concentrations of carboxymethyl chitosan (1 to 4 g L^−1^) promoted seedling growth in *Populus davidiana*, while concentrations equal to or higher than 6 g L^−1^ had inhibitory effects [59]. In grapevine (*Vitis vinifera* L.), a 1% chitosan formulation (Biochikol 020 PC) increased internode number by 33%, shoot length by 71%, and shoot number by 29% under drought stress [58]. These enhancements reflect a coordinated increase in both segmented and total shoot growth. Similar trends were observed in *Lantana camara* L., where 0.1 mg L^−1^ high molecular weight chitosan increased main shoot length to 3.95 cm and lateral shoot length to 0.29 cm, though higher concentrations suppressed elongation and reduced shoot number [39]. In *Vitis vinifera* seedlings, an optimal chitogel concentration of 1.75% (v/v) increased both shoot length and node number, whereas concentrations equal to or greater than 2.0% showed toxic effects [65]. These findings emphasize that internode elongation is not only species dependent but also highly sensitive to chitosan dosage and environmental context, further supporting the importance of medium composition and dose optimization during ex-vitro adaptation.

### SPAD value

SPAD values were significantly influenced by both the chitosan treatments (*p* < 0.05). In the black sand medium, the highest SPAD value (28.80) was recorded with the application of 30 mg L^−1^ chitosan, followed by the control group (27.65). In the cocopeat medium, SPAD values of 28.98 and 28.43 were obtained with 15 mg L^−1^ and 30 mg L^−1^ chitosan, respectively. These results demonstrate that SPAD values varied depending on the type of growing medium. In the cocopeat medium, both 15 and 30 mg L^−1^ chitosan treatments resulted in similarly high SPAD values. However, in black sand, 30 mg L^−1^ chitosan markedly enhanced SPAD readings compared to 15 mg L^−1^ (26.50), indicating a more pronounced dose effect. When medium means were considered, cocopeat showed a slightly higher average SPAD value (28.71) than black sand (27.65). Based on application means, 30 mg L^−1^ chitosan yielded the highest SPAD value overall (28.61), while the lowest was observed with 15 mg L^−1^ chitosan in black sand (26.50) (Table 1).

Numerous studies have demonstrated that chitosan applications can enhance stress tolerance during the acclimatization phase by promoting the accumulation of photosynthetic pigments in plants. Górnik et al. [58] reported that Biochikol 020 PC, a commercial chitosan product, significantly increased leaf chlorophyll content in grapevines grown under both optimal (32% volumetric water content, VWC) and drought conditions (12% VWC), with the highest improvements observed at 0.5% and 1% concentrations, respectively. In tomato plants, a 150 mg L^−1^ chitosan treatment resulted in the highest SPAD value (67.33), indicating its potential to mitigate the adverse effects of salinity stress [56]. In *Mentha piperita*, the application of 80 mg L^−1^ irradiated chitosan increased total chlorophyll by 21.7%, carotenoids by 27.4%, and chlorophyll fluorescence by 16.3% [53]. Similar results have been reported in species such as *Stevia rebaudiana* [68], *Musa acuminata* [28], *Prunus davidiana* [59], *Lactuca sativa* [61], and *Basella rubra* [60], where chitosan enhanced chlorophyll a and b, carotenoids, and total pigment levels [58]. These findings, supported by SPAD and chlorophyll fluorescence measurements, confirm the beneficial effects of chitosan on photosynthetic performance. The underlying mechanisms for these positive effects include the regulation of growth parameters, enhancement of reactive oxygen species (ROS) scavenging capacity, and the modulation of stomatal activity to reduce transpiration losses [40, 61, 67, 69, 70]. During acclimatization, the observed increase in pigment levels in plants transferred from in vitro to ex-vitro conditions facilitates the recovery of photosynthetic capacity and improves adaptation to environmental conditions [28, 71]. However, the efficacy of chitosan may vary depending on its concentration, molecular weight, and formulation. Some studies have reported growth-inhibitory effects at higher concentrations, particularly above 2 g L^−1^ [57, 59] Therefore, determining the optimal dose of chitosan during the ex-vitro adaptation stage is essential for maintaining plant viability and physiological balance.

### Leaf area (cm²)

Leaf area was significantly affected by both chitosan treatments and the type of growing medium (*p* < 0.05). Measurements were performed on plantlets that survived the adaptation phase. In the cocopeat medium, the largest leaf area (25.43 cm²) was recorded with the application of 15 mg L^−1^ chitosan, followed by 30 mg L^−1^ chitosan (24.76 cm²). In contrast, the leaf area values obtained in the black sand medium were considerably lower, with 12.59 cm² and 3.15 cm² corresponding to 15 mg L^−1^ and 30 mg L^−1^ chitosan applications, respectively. These findings indicate that the cocopeat medium provided more favorable conditions for leaf area development at both chitosan concentrations. In the black sand medium, however, the leaf area was significantly limited, particularly at the higher dose. When medium means were considered, cocopeat exhibited a substantially larger average leaf area (25.10 cm²) compared to black sand (7.87 cm²). Based on application means, 30 mg L^−1^ chitosan resulted in the highest overall mean leaf area (19.01 cm²), although this effect was largely attributed to the values obtained in the cocopeat medium (Table 1).

The positive effect of chitosan on leaf area was more evident in the cocopeat medium, a result that aligns with trends observed in previous in vivo and in vitro studies. This can be attributed to cocopeat’s high water-holding capacity and its ability to provide nutritional support. For instance, in *Lactuca sativa*, soil-applied chitosan at concentrations of 0.05–0.15% (w/w) increased leaf area from 674 cm² to as much as 856 cm², while a higher concentration of 0.30% resulted in a reduction in leaf size [61]. Similarly, in *Basella alba*, foliar application of 75 ppm chitosan enhanced leaf area, whereas at 100 ppm this increase diminished [47]. In *Lycopersicon esculentum*, foliar spraying with 0.75 mg mL^−1^ chitosan resulted in an approximately 11% increase in leaf area under normal conditions [67]. In a separate study, application of 150 mg L^−1^ chitosan under salinity stress significantly improved both leaf number and area [56]. In *Prunus davidiana*, application of 2 g L^−1^ carboxymethyl chitosan resulted in a 28.5% increase in leaf biomass, while doses ≥ 6 g L^−1^ reversed this effect [59]. In *Solanum tuberosum*, in vitro application of 500 mg L^−1^ soluble chitosan improved acclimatization and minituber production under ex-vitro conditions, indirectly reflecting improved leaf development and physiological status [57]. The effects related to substrate selection further reinforce these findings. In *Fragaria* × *ananassa*, cocopeat provided the best environment for acclimatization, leading to the highest survival rate, leaf number, and average leaf area due to its high organic carbon, nitrogen, and moisture content [51]. In *Musa acuminata*, chitosan treatments significantly enhanced all growth parameters, including leaf number, when plants were transferred to pots containing a mixture of cocopeat and farmyard manure [28, 40]. Similarly, in *Dendrobium* Shavin White, chitosan-enriched media containing EFB and peat-based mixtures produced the best results in terms of leaf length and number [55]. These findings highlight that the effectiveness of chitosan is not solely dependent on its concentration but is also influenced by the physicochemical properties of the substrate. When combined with growing media that ensure sufficient moisture and nutrient availability, chitosan can more effectively support leaf development.

### Hierarchical cluster analysis (HCA)

The heatmap analysis revealed clear clustering patterns among the chitosan treatments, reflecting their distinct effects on plantlet development parameters. In terms of survival rate, all chitosan treated groups showed considerably higher values, emphasizing the role of chitosan in improving plantlet viability during adaptation. The grouping also highlighted that survival was notably enhanced regardless of growing medium when chitosan was applied.

**Fig. 3:**
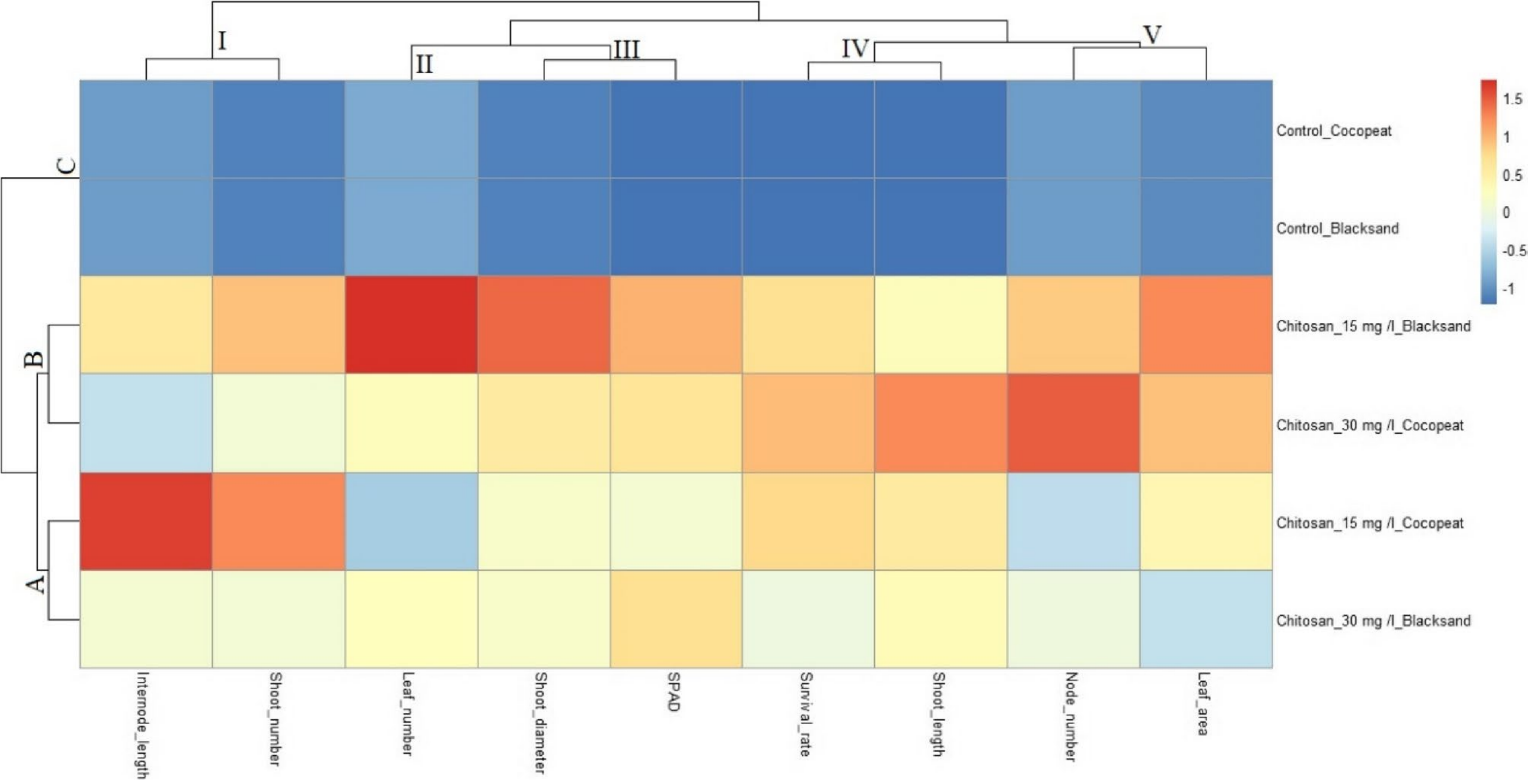
Heatmap for morphological parameters evaluated at the adaptation stage in micropropagation of Fercal grapevine rootstock. Control and chitosan treatments (15 and 30 mg L^−1^) were applied in two different growing media (Cocopeat and Black sand). **A**, **B**, and **C** represent clusters of treatment combinations, while I to V indicate the groups of morphological parameters examined, including internode length, shoot number, leaf number, shoot diameter, SPAD value, survival rate, shoot length, node number, and leaf area. Clustering was based on z-score normalized values

For the remaining morphological and physiological traits, comparisons among chitosan treatments showed that the combination of 30 mg L^−1^ chitosan with cocopeat resulted in the most favorable outcomes for internode length, shoot number, shoot length, and shoot diameter, indicating enhanced vegetative growth. Moreover, SPAD values and leaf area were also higher under cocopeat based applications, particularly at higher chitosan concentration. Among the treatments, 15 mg L^−1^ chitosan in black sand uniquely led to the highest leaf number, whereas the maximum node number was observed in 30 mg L^−1^ chitosan with black sand, pointing to a dose and medium dependent response. Overall, chitosan treatments, especially those combined with cocopeat, were associated with superior plantlet performance across most parameters (Figs. 2 and 3).

The heatmap analysis indicated that chitosan treatments, particularly in cocopeat, significantly enhanced overall plantlet performance. The combination of 30 mg L^−^¹ chitosan with cocopeat promoted internode elongation, shoot development, and higher SPAD and leaf area values, reflecting improved vegetative growth and physiological status. These effects are likely attributed to the synergistic interaction between chitosan and the favorable properties of cocopeat, such as aeration and moisture retention [49, 63, 72]. Meanwhile, black sand treatments revealed that chitosan’s impact on leaf and node number was dose- and substrate-dependent, highlighting the importance of application context.

### Principal component analysis (PCA)

**Fig. 4:**
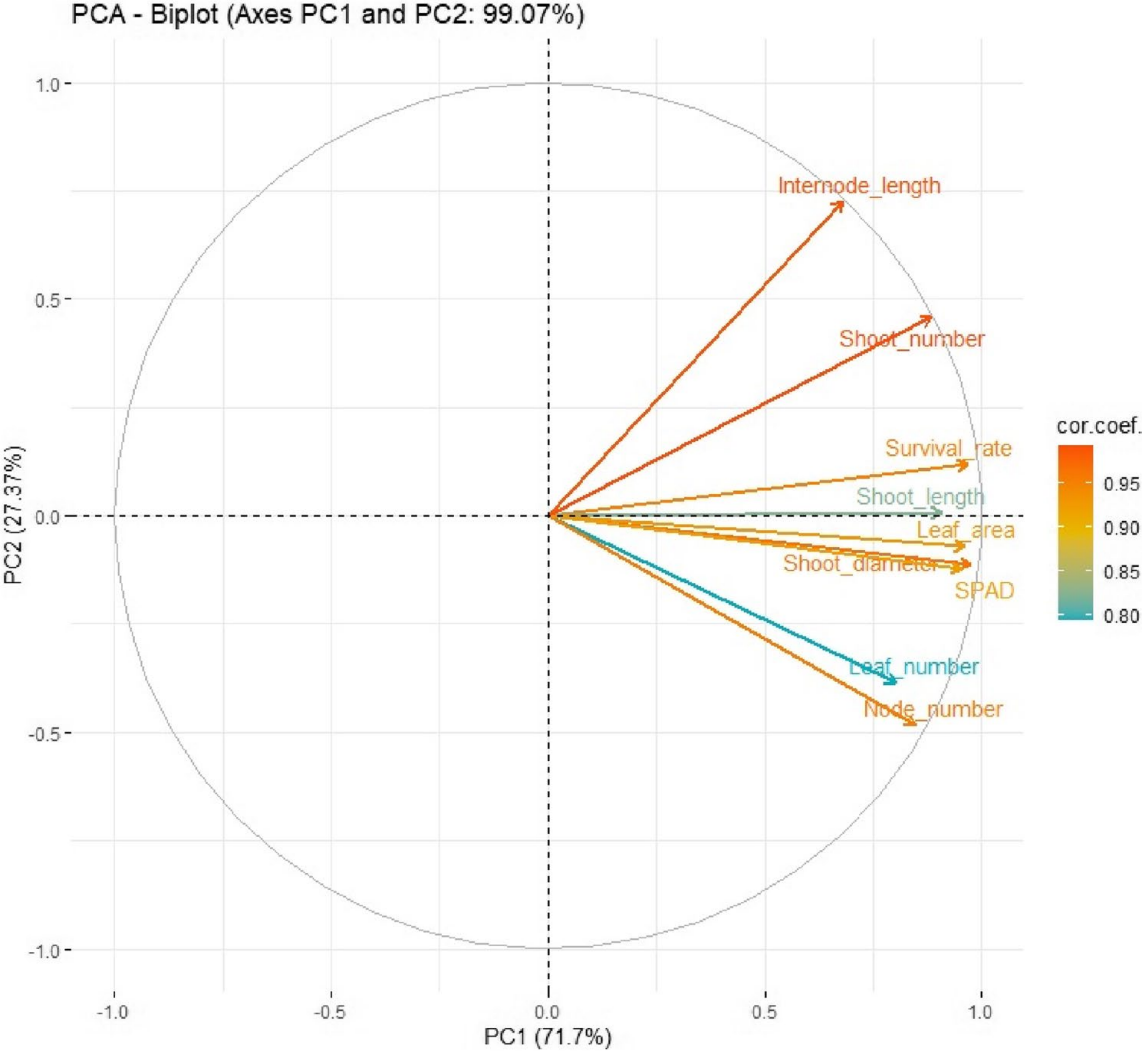
PCA biplot showing the relationships among morphological parameters measured during the adaptation stage of Fercal rootstock. PC1 and PC2 explain 99.07% of the total variation. Arrows represent the direction and strength of correlation between each trait and the principal components

The PCA results revealed that parameters such as internode length, shoot number, shoot length, and survival rate are strong indicators of plant performance during the adaptation stage. The vectors of these traits are oriented in similar directions on the biplot, suggesting that they tend to increase or decrease together, and may therefore represent similar physiological responses. In contrast, leaf number and node number are oriented differently and show relatively lower correlations with the other parameters. This indicates that they may be governed by distinct growth factors or developmental mechanisms compared to the other morphological traits (Fig. 4).

The PCA analysis demonstrated that internode length, shoot number, shoot length, and survival rate clustered closely, suggesting a coordinated response that collectively reflects plantlet vigor during adaptation. These traits, associated with active shoot development, may be influenced by similar physiological processes such as hormonal balance, improved photosynthesis, and resource allocation [67, 73]. In contrast, leaf and node numbers contributed less to overall variance, implying they may be regulated by separate developmental cues Górnik et al. [58]. This separation highlights the complexity of plant response patterns and emphasizes the value of multivariate approaches in identifying key indicators of adaptation success [22, 74].

## Conclusion

This study demonstrated that both the type of growing media and the concentration of foliar-applied chitosan significantly influenced the adaptation success and vegetative development of Fercal rootstock. The highest survival rate was achieved with 30 mg L^−1^ chitosan in black sand, suggesting that the combination of an inert and well-aerated substrate with the bio-stimulant properties of chitosan may have created a more favorable environment for plantlet adaptation. Conversely, the cocopeat medium, particularly when combined with 15 mg L^−1^ chitosan, supported superior shoot elongation, leaf size and SPAD values, indicating that moderate doses of chitosan are more effective in nutrient-rich substrates with favorable moisture retention.

Chitosan treatments generally improved shoot development, stem diameter, chlorophyll content and leaf expansion, with their effects being more pronounced in substrates that either limited nutrient availability such as black sand or enhanced physiological uptake such as cocopeat. The heatmap and PCA analyses further revealed that parameters including internode length, shoot number and survival rate were strongly correlated and served as reliable indicators of plantlet vigor during adaptation.

In addition, the practical applicability of this approach is supported by the ease of sterilization of inert substrates like black sand. Such materials can be efficiently sterilized using autoclaving, which ensures aseptic conditions during the adaptation phase. This makes them suitable for use in tissue culture systems where microbial contamination must be minimized. Therefore, further investigations into the interaction between chitosan concentrations and substrates with distinct physical and chemical characteristics such as cocopeat and black sand are essential.

These findings provide valuable insights for optimizing clonal propagation strategies, particularly for rootstocks intended for cultivation in calcareous or otherwise challenging soils. Future research should aim to identify optimal chitosan dosages tailored to different species and genotypes to fully exploit its potential under various acclimatization conditions.

## Data Availability

The data supporting the findings of this study are available from the corresponding author.

## Abbreviations

ANOVA: Analysis of Variance
BAP: 6-Benzylaminopurine
CaCO_3_: Calcium Carbonate
CF: Coconut Fiber
CRH: Carbonized Rice Husk
DAS: Days After Spraying
EFB: Empty Fruit Bunch
HCA: Hierarchical Cluster Analysis
IBA: Indole-3-Butyric Acid
MS: Murashige and Skoog
NaOCl: Sodium Hypochlorite
PCA: Principal Component Analysis
ROS: Reactive Oxygen Species
SPAD: Soil Plant Analysis Development
TS: Turkish Standard
VIVC: Vitis International Variety Catalogue
v/v: Volume per Volume
VWC: Volumetric Water Content
w/v: Weight per Volume
